# Effect of performance-based financing on health service delivery: a case study from Adamawa state, Nigeria

**DOI:** 10.1093/inthealth/ihaa026

**Published:** 2020-06-12

**Authors:** Ryoko Sato, Abdullahi Belel

**Affiliations:** Department of Global Health and Population, Harvard T.H. Chan School of Public Health, 90 Smith Street, Boston, MA, 02120 USA; Yola, Adamawa State, Nigeria

**Keywords:** performance-based financing, health service delivery, northern Nigeria

## Abstract

The Nigeria State Health Investment Project (NSHIP) was implemented in three Nigerian states between 2013 and 2018. Under the NSHIP, some local government areas were randomly assigned to Performance-Based Financing (PBF) intervention while others received decentralized facility financing (DFF) for comparison. This article evaluates the effect of PBF compared with DFF on health service delivery indicators in Adamawa state, under this quasi-experimental design, using the difference-in-differences technique. The analysis used health facility monthly data collected by the Health Management Information System through the District Health Information Software 2 (DHIS2). The PBF intervention group significantly increased the quantity of most of its service delivery indicators, such as antenatal care visits and deliveries by skilled personnel compared with the comparison group (DFF) after the introduction of NSHIP, although the baseline level of service delivery between PBF and DFF health facilities was statistically identical prior to the introduction of the intervention. We also conducted robustness check analysis to confirm the effect of PBF. Overall, we found a significant positive effect of PBF on most service delivery outcomes, except full vaccinations and post-natal care. One important policy implication is that we should carefully use PBF for targeted indicators.

## Introduction

Although Nigeria is an economic giant in sub-Saharan Africa,^1^ it lags behind many other African countries in terms of health outcome indicators. For example, the mortality rate in 2017 of children <5 y of age was 100 per 1000 live births, which is one of the highest in the world.^2^ Low health outcome indicators can be attributed to low health service utilization. For example, the percentage of births assisted by a skilled birth attendant is only 43% and full immunization coverage is 23%.^3^

While demand-side barriers are found to be contributing factors to low health service utilization,^4^ supply-side barriers such as unqualified health workers, staff absenteeism, inadequate staff, limited operating hours and informal payments also have been found to contribute significantly to the low level of health service utilization.^5^^,^^6^ Strengthening the health system could potentially improve service delivery mechanisms, which could lead to improved health outcomes. One popular approach is called performance-based financing (PBF). This approach intends to improve the service delivery mechanism in the health sector by providing financial incentives to health service providers based on their performance.^7^ Many developing countries have been applying this innovative financing method to strengthen their health systems.^8^ PBF is considered an innovative way to improve service quality. Traditionally health facilities are financed independent of their performance, which can lead to low quality of service delivery.^9^ More than 50 academic papers have evaluated the effectiveness of PBF.^10^ Evidence of its effectiveness is mixed.^11^ For example, Basinga et al.^12^ studied one of the early PBF programs in Africa using a randomized controlled trial. They found that PBF improved both the quantity and quality of maternal and child health service provision in Rwanda. On the other hand, de Walque et al.^13^ found that there was no difference in many indicators between the PBF group and the control group, among which the incentive was not linked to performance.

This article evaluates the effect of the experimental PBF program implemented in Nigeria on various indicators around maternal and child health service utilization, with a rigorous econometrics tool. We particularly focus on Adamawa state, located in the northeastern zone of Nigeria. Broadly, health outcome and utilization indicators are in general poorer in northern Nigeria than in southern Nigeria. For example, facility deliveries are much lower in the northeast (25%) than in the south (82%). Only 25% of births in the northeast are attended by a skilled provider, compared with 85% in the south.^14^ One of the reasons for the poorer health indicators in the north is poor healthcare financing.^15^ However, because public health facilities are the main providers of healthcare for people in the north, strengthening the health system is one of the priorities in northern Nigeria.

## Methodology

### Intervention: Nigeria State Health Investment Project (NSHIP)

With sponsorship from the World Bank, the Nigerian government initiated the NSHIP in 2012, with the general objective of improving the quantity and quality of services delivered in primary healthcare. The NSHIP was implemented in three states initially: Adamawa, Nasarawa and Ondo. In this study, we focus on Adamawa. 

Under the NSHIP, two different payment schemes were implemented to evaluate their effectiveness on service delivery. One was the performance-based financing (PBF) scheme and the other was the decentralized facility financing (DFF) scheme. Under PBF, health facilities (HFs) received a quarterly payment based on the quantity of services delivered. The type of service, which was the basis of the payment, was predetermined. Examples of the types of services included outpatient consultation, complete vaccination cases, tetanus-toxoid vaccination of pregnant women, post-natal care (PNC) consultation, antenatal care (ANC) consultation, family planning services and institutional delivery. Each health facility was paid a certain price for a given service (unit price). The monetary incentives were based on the quantity of services provided, multiplied by the unit price. These funds were used for operational costs and bonuses for health workers of 50% each. Operational costs included the costs for maintenance and repair, as well as drugs and consumables. For detailed information on the incentives for each service, see Kandpal et al.^16^

In contrast, under DFF, health facilities received a certain payment, regardless of the quantity of services delivered. The payments under DFF were calculated to be equal to the average amount of funds for only operational costs earned by the PBF HFs, as DFF HFs were not allowed to use funds to pay health staff bonuses. Thus, by design, the amount DFF HFs received was half the amount earned by PBF HFs.

**Figure 1.**
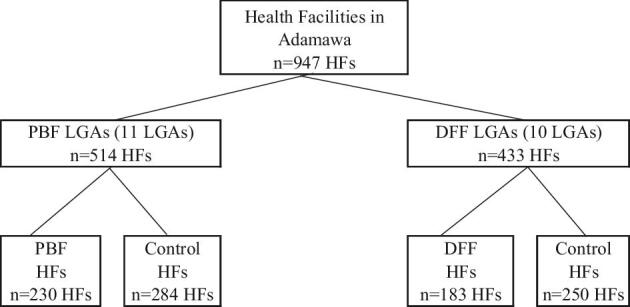
Distribution of HFs by treatment status.

In Adamawa state, the treatment was randomly assigned to each local government area (LGA). Adamawa has 21 LGAs, 11 of which received PBF and the remaining 10 received DFF. The total number of health facilities that received either PBF or DFF was 445. There were a total of 947 health facilities in the state, which were recorded in the data portal called District Health Information Software 2 (DHIS2). About half of the health facilities in Adamawa state did not receive either PBF or DFF and operated as ‘business as usual’, thus they constituted the control group. The health facilities in the control group were those that did not have a sufficient level of functionality in terms of infrastructure and personnel. Figure 1 presents the distribution of health facilities by treatment status.

### Data

We used the health facility–level data collected by the Health Management Information System through the DHIS2. The DHIS2 records monthly information on the quantity of various health services provided by all the health facilities in Adamawa state before and after the introduction of the NSHIP. To identify health facilities in each category (PBF, DFF and control) we compared two sets of health facilities. One was the census list of all the health facilities in Adamawa state, which we obtained from DHIS2 data. The other data source was the restricted list of health facilities, either PBF or DFF, which is publicly available online.^17^ If a health facility in the DHIS2 data was not listed in the restricted data from the PBF portal, which meant that the health facility did not receive PBF or DFF, then it was a control HF.

### Outcomes

We focused on seven outcomes for health services. All the outcomes were measured as quantities, i.e. how many times each health facility provided a particular health service per month. Our seven health service indicators included ANC visit, normal delivery administered, delivery services administered by skilled personnel, full vaccinations in children, outpatient care visit, PNC visit and the third dose of tetanus-toxoid vaccination (TT3) administered. For each outcome we had the information for each health facility for a particular month of the year. We selected these seven indicators because they were important maternal and child health outcomes.

### Statistical analysis

Using the DHIS2 data for health facilities, we evaluated the effect of the PBF intervention compared with DFF on the quantity of various health services provided at health facilities. To do so, we employed the difference-in-differences (DiD) approach. First, we compared the change in the outcome variables between PBF HFs and DFF HFs before and after the introduction of the NSHIP using the following regression framework:

(1) \begin{equation*} {y_{it}} = \ \alpha + {\beta _1}PB{F_{it}} + {\beta _2}{{After}_{it}} + {\beta _3}PBF\_{{After}_{it}} + {v_i} + {\varepsilon _{it}} \end{equation*}

where ${y_{it}}$ is an outcome variable, service provision at health facility *i* at time *t*. $PB{F_{it}}$ is a dummy variable that indicates whether the health facility was assigned to PBF treatment (PBF HFs). The comparison group DFF HFs. ${{After}_{it}}$ is a dummy variable that indicates whether the NSHIP had been introduced to health facility *i* at time *t.* Most LGAs started to enrol in the NSHIP in July 2014 or January 2015, although introduction of the NSHIP differed by LGA. ${{After}_{it}}$ is 0 before introduction of the NSHIP and 1 after introduction. The difference in timing of the introduction of the NSHIP was accounted for in this dummy variable. $PBF\,\,{{After}_{it}}$ is an interaction term between *PBF* and *After*. We used the LGA fixed effect, *v*, to control for LGA-specific characteristics. Because assignment of the treatment (PBF or DFF) was at the LGA level, once we introduced the LGA-level fixed effect, the variable $PB{F_{it}}$ was dropped from the analysis due to perfect multicollinearity.

The main analysis compared the effectiveness of PBF intervention to that of DFF on various outcomes, restricting the sample to health facilities that were assigned to either PBF or DFF, eliminating control health facilities, which were not assigned to either group.

**Figure 2.**
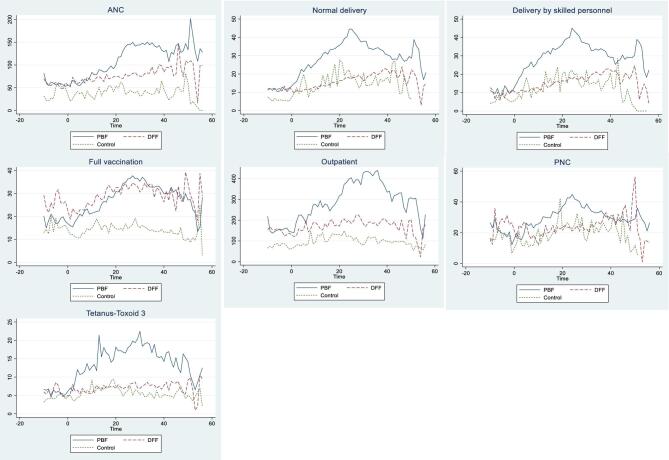
Time trend of health service delivery (PBF vs DFF). The total number of HFs for the analysis is 932. The sample includes HFs that are either PBF, DFF or controls. Time 0 indicates introduction of the NSHIP in each LGA.

${\beta _1}$ identifies differences between PBF HFs and DFF HFs before introduction of the NSHIP. However, as mentioned above, with the LGA fixed effect, ${\beta _1}$ is dropped automatically. ${\beta _2}$ identifies the time trend after initiation of the NSHIP among DFF HFs, as compared with before introduction of the NSHIP. ${\beta _3}$ is the coefficient of interest. It captures the difference-in-differences estimator of the effect of PBF. This DiD estimation strategy is valid only under the assumption that the time trend of the outcome would have been the same between PBF HFs and DFF HFs in the absence of the NSHIP intervention. This parallel trend assumption prior to the introduction of NSHIP seems to hold (Figure 2).

We hypothesize that the PBF program improved service delivery more than the DFF program. In other words, we expect ${\beta _3} > 0$.

Second, we evaluated the effectiveness of the PBF intervention by comparing PBF HFs and control HFs. Because assignment of the treatment, either PBF or DFF, was done at the LGA level, we focused only on PBF LGAs. We used the same identification strategy as in equation (1) with the LGA fixed effect, with control HFs being the comparison group. In this analysis, ${\beta _1}$ does not drop, because there is a variation in the treatment assignment, either PBF or control, within each health facility.

Finally, to confirm the rigorous effect of the PBF intervention, we conducted a placebo test. Using equation (1), we evaluated the difference in the provision of health services between health facilities in the control group (control HFs) within PBF LGAs and health facilities in the control group (control HFs) within DFF LGAs. In the absence of the NSHIP, heath facilities in PBF LGAs should have similar characteristics to ones in DFF LGAs, unless they were systematically different. If the randomization at the LGA level worked well, we expect that ${\beta _3}$ is not different from 0.

## Results

Of 947 health facilities (HFs) in Adamawa state, we used 932 facilities for the analysis. The remaining 15 health facilities did not have sufficient data to be included in the analysis.

Table 1 presents the baseline level of health services provision according to the intervention status—PBF HFs (column 1), DFF HFs (column 2) and control HFs (column 3)—before introduction of the NSHIP. For example, the average number of ANC service provisions per month in PBF HFs is 59.45, while it is 62.60 and 35.00 under DFF HFs and control HFs, respectively.

**Table 1.** Baseline health service delivery

Health service	PBF	DFF	Control	Difference (SE) PBF vs DFF	Difference (SE) PBF vs control
	(1)	(2)	(3)	(4)	(5)
ANC	59.45	62.60	35.00	−3.14 (10.55)	24.46 (12.29)*
Normal delivery	12.24	13.16	6.82	−0.93 (1.86)	5.42 (1.28)***
Delivery by skilled personnel	9.90	12.95	6.42	−3.05 (2.32)	3.48 (1.63)**
Full vaccination	19.27	24.67	15.03	−5.39 (3.31)	4.24 (2.03)*
Outpatient	158.02	152.43	75.77	5.60 (22.57)	82.25 (15.75)***
PNC	23.29	26.42	19.38	−3.13 (7.87)	3.91 (3.87)
Tetanus-toxoid 3	6.80	6.44	4.66	0.36 (1.13)	2.14 (1.03)*

The total number of HFs for the analysis is 932. The sample includes HFs that are either PBF, DFF or controls. The difference is with LGA clustered standard error (SE).

*Significant at 10%, **significant at 5%, ***significant at 1%.

All seven outcome indicators are balanced between PBF and DFF HFs. None of the outcome variables at baseline is significantly different between the PBF and DFF HFs (column 4). In contrast, we found a significant difference in outcome indicators between PBF and control HFs (column 5). The baseline health service provision is generally higher among PBF HFs than among control HFs, because control HFs were those that did not have sufficient functionality in terms of infrastructure and personnel.

Figure 2 presents the trend in the quantity of services provided over time, separately among PBF, DFF and control HFs. The horizontal axis presents the time. At time 0, the NSHIP was introduced in each LGA. The timing of the NSHIP's introduction differs by LGA. Any time before time 0 is the pre-intervention period for each LGA. The time trend of the quantity of service provision among PBF HFs and DFF HFs before introduction of the NSHIP was mostly balanced (Table 1 column 4), while the quantity was significantly lower among control HFs (Table 1 column 5).

Immediately after introduction of the NSHIP, the quantity of the provision of most services under PBF increased more than under DFF or controls (Figure 2). The exception is the full vaccination cases. Although the number of full vaccination cases seems much higher under PBF than controls, it is similar to the number under DFF.

Table 2 presents the main result of the effect of the PBF intervention on various health services provision by comparing PBF HFs and DFF HFs using a DiD approach. For five main outcomes out of seven, the PBF intervention significantly increased the quantity of service provision as compared with DFF after introduction of the NSHIP (‘PBF After’ in Table 2). The number of ANC cases increased by about 30 under PBF relative to DFF (column 1), the number of normal deliveries by 12 cases (column 2), the number of deliveries by skilled personnel by 18 cases (column 3), the number of outpatients by 106 cases (column 5) and the number of third-dose administrations of tetanus-toxoid by 8 cases (column 7). Among DFF HFs, the quantity of provision of some services, such as ANC (column 1), normal delivery (column 2), full vaccination (column 4) and outpatients (column 5), also significantly increased after introduction of the NSHIP (‘After’ in Table 2).

**Table 2.** Effect of PBF on health service delivery (DiD)

Effect	ANC	Normal delivery	Delivery by skilled personnel	Full vaccination	Outpatient	PNC	Tetanus-toxoid 3
	(1)	(2)	(3)	(4)	(5)	(6)	(7)
LGA=PBF	0.000	0.000	0.000	0.000	0.000	0.000	0.000
	(.)	(.)	(.)	(.)	(.)	(.)	(.)
After	14.200*	5.504**	2.522	5.944***	32.177**	−2.164	0.634
	(7.332)	(2.299)	(1.938)	(1.303)	(13.515)	(4.767)	(0.748)
PBF After	29.891***	12.386***	17.759***	3.800	105.904***	9.927	8.367***
	(8.969)	(2.664)	(2.481)	(2.634)	(24.453)	(5.821)	(1.735)
Constant	65.976***	13.162***	12.561***	21.649***	167.292***	24.787***	6.578***
	(3.739)	(1.109)	(1.122)	(1.214)	(11.061)	(2.478)	(0.827)
N	16 999	15 688	14 039	14 938	16 971	13 648	9450
r^2^	0.014	0.006	0.040	0.008	0.017	0.003	0.028

The sample includes HFs that are either PBF or DFF, excluding controls. The comparison group is LGA under DFF. The analysis controls for LGA fixed effect with LGA clustered standard error.

*Significant at 10%, **significant at 5%, ***significant at 1%.

Table 3 compares PBF and control HFs (‘business as usual’) in PBF LGAs as a robustness check analysis. The PBF intervention produced an increase in the quantity of services provided for five indicators out of seven in PBF HFs as compared with the control HFs. For example, after introduction of the PBF program, the number of ANC cases increased by 33 in PBF HFs relative to control HFs (column1), the number of deliveries assisted by skilled personnel by 9 (column 3), the number of full vaccination cases by 5 (column 4), outpatients by 80 (column 5) and the number of third doses of tetanus-toxoid by 3 (column 7).

**Table 3.** Effect of PBF on health service delivery (robustness: PBF vs control in PBF LGA)

Effect	ANC	Normal delivery	Delivery by skilled personnel	Full vaccination	Outpatient	PNC	Tetanus-toxoid 3
	(1)	(2)	(3)	(4)	(5)	(6)	(7)
HF=PBF	35.956***	10.632***	6.476*	8.197***	124.749***	5.678	4.401***
	(8.170)	(2.506)	(3.473)	(1.756)	(27.704)	(5.405)	(1.356)
After	−4.956	5.276**	−2.970	−1.160	9.048	−3.023	1.473
	(4.765)	(2.298)	(3.434)	(1.091)	(25.059)	(4.825)	(1.078)
PBF After	33.171***	1.934	8.686*	4.504***	80.499***	4.393	3.154**
	(7.775)	(2.461)	(3.977)	(1.111)	(23.039)	(4.011)	(1.343)
Constant	42.740***	11.636****	17.511***	15.859***	97.896***	23.791***	5.432***
	(8.252)	(2.437)	(3.106)	(1.791)	(25.696)	(6.128)	(1.377)
N	13 176	11 156	10 463	13 083	15 815	10 394	7469
r^2^	0.083	0.052	0.061	0.085	0.119	0.014	0.050

The sample includes HFs that are in a PBF LGA, either PBF HFs or control HFs. The comparison group is control HFs. The analysis controls for LGA fixed effect, with LGA clustered standard error.

*Significant at 10%, **significant at 5%, ***significant at 1%.

Table 4 compares DFF and control HFs in DFF LGAs as another robustness check analysis. The DFF scheme produced an increase in the quantity of services provided for two indicators out of seven in DFF HFs as compared with the control HFs. After introduction of the DFF program, the number of deliveries assisted by skilled personnel increased by six (column 3) and the number of full vaccination cases by two (column 4).

**Table 4.** Effect of DFF on Health Service Delivery (Robustness: DFF vs. Control in DFF LGA)

Effect	ANC	Normal delivery	Delivery by skilled personnel	Full vaccination	Outpatient	PNC	Tetanus-toxoid 3
	(1)	(2)	(3)	(4)	(5)	(6)	(7)
HF=DFF	18.661	5.278	0.585	10.892***	72.683**	6.514	2.443***
	(23.357)	(4.261)	(7.068)	(2.129)	(24.768)	(6.023)	(0.518)
After	−8.576	3.102	−3.038**	0.008	7.088	−1.179	0.149
	(9.787)	(2.886)	(1.196)	(0.733)	(7.688)	(1.936)	(0.222)
DFF After	21.741	0.338	5.855**	2.290*	23.971	0.356	0.282
	(12.004)	(3.258)	(1.835)	(1.155)	(13.355)	(3.582)	(0.727)
Constant	45.496**	8.622**	12.887**	15.678***	74.572***	17.827***	4.348***
	(19.174)	(3.340)	(5.402)	(1.285)	(18.106)	(4.263)	(0.279)
N	11 737	8945	8324	11 083	11 999	7371	5878
r^2^	0.029	0.001	0.019	0.039	0.042	0.005	0.022

The sample includes HFs that are in a DFF LGA, either DFF HFs or control HFs. The comparison group is control HFs. The analysis controls for LGA fixed effect, with LGA clustered standard error.

*Significant at 10%, **significant at 5%, ***significant at 1%.

Table 5 presents the result of a placebo test. We tested whether health facilities in PBF LGAs and DFF LGAs would have had different characteristics over time in the absence of the NSHIP by comparing control HFs in PBF LGAs and control HFs in DFF LGAs. The effect of the NSHIP program on all of the indicators of service provision was not significantly different between control HFs in PBF LGAs and control HFs in DFF LGAs (‘PBF After’ in Table 4).

**Table 5.** Effect of PBF on health service delivery (placebo test: among control HFs)

Effect	ANC	Normal delivery	Delivery by skilled personnel	Full vaccination	Outpatient	PNC	Tetanus-toxoid 3
	(1)	(2)	(3)	(4)	(5)	(6)	(7)
LGA=PBF	0.000	0.000	0.000	0.000	0.000	0.000	0.000
	(.)	(.)	(.)	(.)	(.)	(.)	(.)
After	−12.659	3.540	4.939	−0.155	13.015	2.064	0.516
	(22.778)	(2.795)	(5.552)	(1.835)	(19.098)	(2.763)	(0.546)
PBF After	13.626	3.939	3.113	0.078	−10.290	−4.286	1.016
	(23.113)	(3.182)	(5.949)	(2.861)	(24.107)	(5.891)	(1.242)
Constant	42.276***	9.324***	7.069**	14.300***	91.840***	19.574***	4.689***
	(10.166)	(1.334)	(2.905)	(1.277)	(9.867)	(2.360)	(0.491)
N	5480	2589	2495	6673	8170	2363	2755
r^2^	0.001	0.005	0.004	0.000	0.000	0.000	0.003

The sample includes HFs that are only control HFs, excluding PBF HFs and DFF HFs. The comparison group is LGA under DFF. The analysis controls for LGA fixed effect, with LGA clustered standard error.

*Significant at 10%, **significant at 5%, ***significant at 1%.

## Discussion

The effect of PBF on the quantity of services delivered was evaluated in the northeastern Nigerian state of Adamawa. 

Overall, PBF was found to be highly effective in increasing the quantity of health service delivery such as ANC, child delivery, PNC and vaccination as compared with DFF. The PBF intervention significantly increased the quantity of most of the service delivery indicators more than DFF after introduction of the NSHIP, while the baseline level of service delivery between PBF and DFF HFs was statistically identical prior to introduction of the intervention. Our robustness check analysis confirms the positive effect of PBF on service delivery by comparing outcomes between PBF HFs and control HFs within PBF LGAs. We also found that DFF HFs are better at increasing service utilization than control HFs within DFF LGAs, but not as much as the increase observed among PBF HFs. Our placebo test reassures us that the increase in the quantity of service provision is due to the introduction of PBF.

Among seven indicators, PBF interventions did not induce a significant increase in the number of cases of full vaccination or PNC as compared with the DFF intervention. The insignificant result of PBF on these two outcomes is consistent with findings from other studies.^12^^,^^18^

Because the service utilization of both full immunization and PNC are extremely limited in northern Nigeria, yet are considered critical to reduce maternal and child health burdens,^18^^,^^19^ it is important to investigate further the potential reasons why the PBF intervention did not influence the full vaccination cases and PNC. For example, only 20% of children in northeastern Nigeria have completed the full vaccination schedule, while >40% of children in southern Nigeria have completed the full schedule.^20^ The rate of PNC visits in northeastern Nigeria is 34.3%, while the southern regions achieve >65%.^21^

Basinga et al.^12^ explained that the low unit price for service delivery causes the insignificant effect of the PBF intervention. However, the average unit price for full vaccination is about 1454 naira (US$1=360 naira as of June 2019) and the price for PNC is 394 naira (Table 6). These unit prices are not as low as that of ANC (294 naira) or the third dose of tetanus-toxoid (197 naira), for which we found a strong positive effect of PBF. Thus the low unit price is unlikely to be causing the insignificant effects of PBF. Basinga et al.^12^ also mentioned that if the baseline immunization rate is already high, then there is little room for improvement. However, as explained above, both full immunization and PNC visits are extremely limited in northern Nigeria. Thus the high-level of baseline health service utilization is unlikely to be causing the insignificant effect of PBF.

**Table 6.** Average unit price (naira)

Healthcare provided	Mean	SD	Minimum	Maximum
ANC	293.5	89	110	585
Normal delivery	2953.7	864	936	4066
Full vaccination	1454.4	461	360	2033
New outpatients	99.8	27	50	135
PNC	394.3	115	180	542
TT	197.0	58	45	271

The unit price is the average price among the available (n = 221) PBF HFs in Adamawa state in the DHIS2 data.

SD: standard deviation.

Future studies should explore reasons why the PBF intervention did not impact the full immunization rate and PNC service. One potential reason why PBF had no effect on full immunization might have been because it involved repeated actions of caregivers’ clinic visits, which made it difficult to monitor.

Despite strong evidence of the effectiveness of the PBF intervention, there is some concern about its sustainability. Although we observed that the quantity of services provided increased right after the introduction of PBF, the quantity of many services delivered, such as the number of child deliveries, outpatients, PNC and third doses of tetanus-toxoid decreased toward the end of the intervention (Figure 2). This inverted-U relationship between the time since the intervention's introduction and the quantity of services provided poses some questions on the sustainability of the PBF intervention. Future work should explore the reasons for this inverted-U relationship. To the authors’ knowledge, there is no study explaining the inverted-U relationship of PBF and health indicators. One potential explanation of the inverted-U relationship is evaluation fatigue.

Our results have an important policy implication. Because PBF does not improve the health service utilization of some indicators in a short-term or sustainable way, we should carefully use the PBF scheme for targeted indicators.

## Limitations

This study has several limitations. First, DHIS2 is an aggregated database that does not contain any patient information. Second, the data also do not contain the location information of each health facility, except the name of the LGA it belongs to. This limited availability of information makes it difficult for the study to analyse the differential effect of PBF on different segments of the population, for example, by wealth level and by access to a health facility. Third, the data incompleteness of the DHIS2, such as missing monthly information on main health indicators as well as missing key information on the characteristics of health facilities, can prevent us from determining a rigorous estimate of the PBF effect. Fourth, the comparison between PBF HFs and control HFs does not rigorously reveal the effect of PBF because the baseline characteristics of control HFs are fundamentally different from those of PBF HFs. Thus the robustness check analysis should be interpreted with caution. Fifth, the mechanisms of PBF's superiority over DFF were not identified under this current research design. PBF was presumably successful either because the total amount of funds available to PBF was double that available to DFF or the incentive structure of PBF worked. Extant studies in other African countries have also pointed out the complexity of the potential mechanisms of PBF.^24^ Sixth, this study only focuses on service utilization, not health outcomes. Although some of the health service indicators we used in the analysis, such as vaccination, are proven to lead to improved health, we should exercise caution in translating our results to policy implications, because the increase in the number of service deliveries might not necessary lead to improvement in the quality of service provision.

## Conclusions

PBF is highly effective in increasing the quantity of health services delivered in Adamawa state, Nigeria. However, the number of full immunizations and PNC cases did not increase due to PBF. Future work should explore why PBF was effective in increasing the delivery of some services but not others.

## References

[1] International Monetary Fund Regional economic outlook. Sub-Saharan Africa: recovery amid elevated uncertainty. Washington, DC: International Monetary Fund; 2019.

[2] United Nations Inter-agency Group for Child Mortality Estimation. Child mortality rate – Nigeria. 2018 Available from: https://childmortality.org/data/Nigeria 10.1371/journal.pone.0101112 PMC4094389 25013954

[3] National Bureau of Statistics, United Nations Children's Fund Multiple Indicator Cluster Survey 2016–17. National Survey Finding Report. Abuja, Nigeria: National Bureau of Statistics and United Nations Children's Fund 2017 Available from: https://www.unicef.org/nigeria/sites/unicef.org.nigeria/files/2018-09/Nigeria-MICS-2016-17.pdf

[4] Ensor T , Cooper S Overcoming barriers to health service access: influencing the demand side. Health Policy Plan. 2004;19(2):69–79. 14982885 10.1093/heapol/czh009

[5] Kyei-Nimakoh M , Carolan-Olah M, McCann TV Access barriers to obstetric care at health facilities in sub-Saharan Africa—a systematic review. Syst Rev. 2017;6(1):110. 28587676 10.1186/s13643-017-0503-x PMC5461715

[6] Adedini SA , Odimegwu C, Bamiwuye O et al. Barriers to accessing health care in Nigeria: implications for child survival. Global Health Action. 2014;7(1):23499. 24647128 10.3402/gha.v7.23499 PMC3957799

[7] Musgrove P Financial and other rewards for good performance or results: a guided tour of concepts and terms and a short glossary. Washington, DC: World Bank; 2011.

[8] Eichler R , AgarwalK, AskewIet al. Performance-based incentives to improve health status of mothers and newborns: what does the evidence show? J Health Popul Nutr. 2013;31(4 Suppl 2):36–47.24992802

[9] Meessen B , Soucat A, Sekabaraga C Performance-based financing: just a donor fad or a catalyst towards comprehensive health-care reform? Bull World Health Org. 2011;89:153–6. 21346927 10.2471/BLT.10.077339 PMC3040374

[10] Craig L Evaluation research on results-based financing. An annotated bibliography of health science literature on RBF indicators for reproductive maternal newborn child and adolescent health. MEASURE Evaluation Working Paper, Carolina Population Center, Chapel Hill, NC 2017.

[11] Paul E , Albert L, Bisala BN'S et al. Performance-based financing in low-income and middle-income countries: isn't it time for a rethink? BMJ Global Health. 2018;3(1):e000664. 10.1136/bmjgh-2017-000664 PMC5859812 29564163

[12] Basinga P , Gertler PJ, Binagwaho A et al. Effect on maternal and child health services in Rwanda of payment to primary health-care providers for performance: an impact evaluation. Lancet. 2011;377(9775):1421–8. 21515164 10.1016/S0140-6736(11)60177-3

[13] De Walque, Damien BCM , Robyn PJ et al. Looking into the performance-based financing black box: evidence from an impact evaluation in the health sector in Cameroon (English). Policy Research Working Paper 8162, Impact Evaluation series. Washington, DC: World Bank Group; 2017 Available from: http://documents.worldbank.org/curated/en/834601502391015068/Looking-into-the-performance-based-financing-black-box-evidence-from-an-impact-evaluation-in-the-health-sector-in-Cameroon. 10.1093/heapol/czab002 PMC12147217 33963406

[14] Lawanson AO , Olanrewaju O Health expenditure and health status in northern and southern Nigeria: a comparative analysis using National Health Account framework. Afr J Health Econ. 2013;2:31–42.

[15] Kandpal E , Loevinsohn BP, Vermeersch CMJ et al. Impact evaluation of Nigeria State Health Investment Project. Washington, DC: World Bank Group; 2019 Available from: http://documents.worldbank.org/curated/en/589301552969360031/Impact-Evaluation-of-Nigeria-State-Health-Investment-Project

[16] National Primary Healthcare Development Agency PBF Portal. Federal Republic of Nigeria. Available from: http://pbfnigeria.org/data.html [accessed 18 June 2019].

[17] National Population Commission, ICF International Nigeria Demographic and Health Survey 2018. Abuja, Nigeria, and Rockville, MD, USA: National Population Commission and ICF International; 2019.

[18] Andre FE , Booy R, Bock HL et al. Vaccination greatly reduces disease, disability, death and inequity worldwide. Bull World Health Org. 2008;86(2):140–6. 18297169 10.2471/BLT.07.040089 PMC2647387

[19] World Health Organization WHO recommendations on postnatal care of the mother and newborn. Geneva: World Health Organization; 2014. 24624481

[20] Paul E , Dramé ML, Kashala J-P et al. Performance-based financing to strengthen the health system in Benin: challenging the mainstream approach. Int J Health Policy Manage. 2018;7(1):35–47. 10.15171/ijhpm.2017.42 PMC5745866 29325401

[21] World Health Organization Regional Office for Africa Atlas of African health statistics 2018: universal health coverage and the sustainable development goals in the WHO African Region. Brazzaville: World Health Organization Regional Office for Africa; 2018 Available from: http://www.aho.afro.who.int/sites/default/files/Atlas%202018-eng_1.pdf.

